# Anti-HMGCR-Antibody-Positive Statin-Induced Myositis: A Pilot Case Series on Treatment with Bempedoic Acid and Immunosuppressive Therapy

**DOI:** 10.3390/antib14030063

**Published:** 2025-07-23

**Authors:** Maurizio Benucci, Riccardo Terenzi, Francesca Li Gobbi, Emanuele Antonio Maria Cassarà, Tommaso Picchioni, Edda Russo, Barbara Lari, Mariangela Manfredi, Maria Infantino

**Affiliations:** 1 Rheumatology Unit, S. Giovanni di Dio Hospital, Azienda USL-Toscana Centro, 50143 Florence, Italy; riccardo.terenzi@uslcentro.toscana.it (R.T.); francesca.ligobbi@uslcentro.toscana.it (F.L.G.); emanueleantonio.cassara@uslcentro.toscana.it (E.A.M.C.); 2 Internal Medicine Unit, S. Giovanni di Dio Hospital, Azienda USL-Toscana Centro, 50143 Florence, Italy; tommaso.picchioni@uslcentro.toscana.it; 3 Clinical Pathology S. Giuseppe Hospital, Azienda USL-Toscana Centro, 50053 Florence, Italy; edda.russo@gmail.com; 4 Immunology and Allergology Laboratory Unit, S. Giovanni di Dio Hospital, Azienda USL-Toscana Centro, 50143 Florence, Italy; barbara.lari@uslcentro.toscana.it (B.L.); mariangela.manfredi@uslcentro.toscana.it (M.M.); maria2.infantino@uslcentro.toscana.it (M.I.)

**Keywords:** anti-HMGCR antibodies, bempedoic acid, statin-induced myositis

## Abstract

Background/Objectives: Immune-mediated necrotizing myopathy (IMNM) is a severe inflammatory myopathy marked by proximal muscle weakness, elevated creatine kinase (CK), and the presence of anti-HMGCR antibodies. Statin exposure is a recognized trigger for anti-HMGCR-positive IMNM, which may persist despite statin withdrawal. This pilot case series explores, for the first time, the use of bempedoic acid—a liver-specific lipid-lowering agent with minimal muscle toxicity—as an alternative to statins in these patients. Methods: We report 10 anti-HMGCR-antibody-positive IMNM patients (6 females, 4 males) previously on statins for primary prevention (8 on atorvastatin, 2 on simvastatin) without prior cardiovascular events. Statins were discontinued at myositis onset. All patients received prednisone and immunosuppressants (methotrexate in 7, mycophenolate in 3), plus bempedoic acid. Anti-HMGCR antibodies were measured using a chemiluminescence method. Results: Their mean anti-HMGCR antibody levels decreased significantly from 390.93 ± 275.22 to 220.89 ± 113.37 CU/L (*p* = 0.027) after 6 months of treatment. Their CK levels dropped from 1278.9 ± 769.39 to 315.1 ± 157.72 IU/L (*p* = 0.001), and aldolase dropped from 11.63 ± 2.18 to 6.61 ± 1.22 U/L (*p* = 0.0001). The mean LDL-C value was 96.1 ± 8.16 mg/dL. No disease recurrence was observed. Autoimmune panels were negative for other myositis-associated and/or -specific antibodies. Conclusions: Bempedoic acid appears to be a safe, effective, and cost-efficient lipid-lowering alternative in statin-intolerant IMNM patients. Larger studies are warranted to confirm its efficacy across different subgroups and to optimize dyslipidemia management in this setting.

## 1. Introduction

Immune-mediated necrotizing myopathy (IMNM), also known as necrotizing autoimmune myopathy (NAM), is a distinct subtype of inflammatory myopathies recognized in recent classification criteria [1,2]. It is characterized by the presence of myositis-specific autoantibodies (MSAs), marked proximal muscle weakness, and significantly elevated creatine kinase (CK) levels, often exceeding the upper limit of the normal values by up to ten times. Histopathological findings typically reveal muscle fiber necrosis with minimal inflammatory infiltrate. IMNM can be classified into three distinct categories based on serological profiles: anti-signal recognition particle (SRP) antibody-positive, anti-3-hydroxy-3-methylglutaryl-coenzyme A reductase (HMGCR) antibody-positive, and seronegative IMNM.

HMGCR was initially identified as a potential autoantigen in IMNM due to observations that several patients developed the condition following statin exposure [3,4,5]. In detail, HMGCR catalyzes the conversion of (3S)-hydroxy-3-methylglutaryl CoA (HMG-CoA) into mevalonic acid, a critical step in the biosynthesis of cholesterol. Statins exert their cholesterol-lowering effects by inhibiting HMGCR’s activity, thereby reducing endogenous cholesterol production [6,7]. While statins are generally well tolerated, up to 25% of patients experience musculoskeletal side effects [6]. These can range from an asymptomatic increase in CK levels to muscle pain, weakness, myositis, and, in severe cases, rhabdomyolysis. Most patients recover spontaneously upon the discontinuation of statin therapy. However, in rare cases, statins may trigger an immune-mediated response that persists even after the drug is withdrawn [8,9,10].

Several mechanisms have been proposed to explain the pathogenesis of IMNM, including genetic predisposition and environmental factors such as vitamin D supplementation. Moreover, among the genetic risk factors, the MHC class II allele HLA-DRB111:01 has been strongly associated with a predisposition to anti-HMGCR-positive IMNM [11,12].

Histologically, muscle biopsies from anti-HMGCR-positive patients typically reveal muscle fiber necrosis, upregulation of MHC class I, and infiltration by the macrophages, supporting an antibody-mediated mechanism of toxicity. These autoantibodies contribute to inflammation, oxidative stress, and muscle atrophy by downregulating interleukin-4 (IL-4) and interleukin-13 (IL-13), thereby impairing myoblast fusion and muscle regeneration. Although complement activation is often present, therapeutic inhibition of terminal complement components—such as with C5 inhibitors—has not proven effective [13].

Once disease control is achieved, glucocorticoids should be tapered to the lowest effective dose, with maintenance therapy using methotrexate and/or rituximab continued for a minimum of two years of stable disease before considering withdrawal of immunosuppression [2].

Currently, the treatment for anti-HMGCR-positive IMNM is indeed primarily based on evidence from case series, observational cohort studies, and expert consensus because randomized controlled trials (RCTs) are lacking in this area. This is a rare disease, and high-quality RCTs are difficult to conduct, so clinical practice relies heavily on observational data and expert recommendations. The 224th ENMC International Workshop in 2016 issued consensus recommendations, advising the initiation of glucocorticoid therapy (either oral or intravenous for severe presentations), in combination with a steroid-sparing immunosuppressant such as methotrexate, azathioprine, or mycophenolate mofetil. Intravenous immunoglobulins (IVIGs) may be added in more severe or refractory cases. If the clinical response remains inadequate after six months, the addition of rituximab is recommended.

There is currently no clear evidence indicating whether the continuation of statins contributes to ongoing disease activity in patients with statin-induced IMNM. Moreover, few studies have evaluated whether switching to a different statin, rather than continuing the same agent, offers a safer or more effective lipid-lowering strategy in these patients. Given that individuals with statin-induced IMNM often have a high baseline risk of cardiovascular disease, the management of dyslipidemia remains a critical component of their overall care.

In the present study, we report, for the first time, a case series of patients diagnosed with statin-associated anti-HMGCR-antibody-positive IMNM receiving a combination of immunosuppressive therapy and bempedoic acid, with a clinical improvement in their symptoms observed, as supported by a review of the current literature [14,15]. In detail, bempedoic acid is an ATP citrate lyase inhibitor that reduces cholesterol synthesis upstream of HMGCR—the enzyme targeted by statins. Like statins, bempedoic acid lowers hepatic cholesterol synthesis and increases LDL receptor expression, enhancing the clearance of LDL cholesterol from the bloodstream [16].

However, unlike statins, bempedoic acid is a pro-drug activated primarily in the liver and not in peripheral tissues such as the skeletal muscle. This tissue-specific activation significantly reduces the risk of muscle-related side effects, making it a potentially safer alternative for lipid-lowering therapy in patients with statin-associated IMNM [17,18].

## 2. Material and Methods

### 2.1. Patients

We retrospectively evaluated our study cohort of 10 patients (6 females and 4 males) affected by IMNM, with a mean age of 62.3 ± 10.8 years. All patients were taking statins (8 with 40 mg/day of atorvastatin, 2 with 20 mg/day of simvastatin) for primary prevention and had no history of cardiovascular events. The number of months of statin treatment was on average 17.1 ± 5.86. The clinical presentation varied between patients, although their CK levels were consistently elevated. After the onset of myositis, statins were discontinued, and treatment with steroids and immunosuppressants was initiated. Specifically, all patients received Prednisone (mean dose: 27 ± 10.12 mg/day) with a progressive decrease. A methotrexate (MTX) dose of 15 mg/week was used in seven patients, while mycophenolate was used in three patients at a dose of 360 mg/day. The interval between the onset of myositis and the start of bempedoic acid inhibitor therapy varied depending on the patient’s symptoms but was generally 3.5 ± 0.53 months. It was important to evaluate the effects of bempedoic acid (180 mg/day) on their LDL levels and muscle safety. In two patients, Ezetinibe at a dose of 10 mg/die was added.

### 2.2. Methods

The determination of anti-HMGCR antibodies was performed using the QUANTA Flash HMGCR chemiluminescence assay (Inova Diagnostics Inc., San Diego, CA, USA). An assay involving a recombinant human HMGCR antigen coupled with paramagnetic beads was performed using the BIO-FLASH system (Biokit S.A., Barcelona, Spain). The BIO-FLASH instrument is a fully automated chemiluminescent immuno-analyzer. The cutoff used was 20 Chemiluminescence Units (CU)/L.

The patients were also evaluated for the following using tests: CK, aldolase, antinuclear antibodies (ANAs), and myositis-associated or -specific antibodies. ANA determination was carried out using IIF on HEp2 cells (Euroimmun AG, Luebeck, Germany), while MAAs and MSAs were detected using the EUROLINE Autoimmune Inflammatory Myopathies lineblot (anti-Mi2, anti-TIF1γ, anti-MDA5, anti-NXP2, anti-SAE1, anti-Ku, anti-PM-Scl, anti-PL7, anti-PL12, anti-EJ, and anti-OJ).

## 3. Results

The demographic, clinical, and laboratory characteristics of the patient cohort are comprehensively summarized in Table 1. The patient group was selected to ensure a homogeneous cohort for the purpose of this study. While the sample size was relatively small, which may have limited the generalizability of the findings, a rigorous statistical analysis was still employed to draw meaningful conclusion. Specifically, Student’s t-test for unpaired data was applied to assessing the statistical significance of the observed changes in biomarkers following the therapeutic interventions.

At the onset of myositis, anti-HMGCR antibody levels were measured to be 390.93 ± 275.22 CU/L on average. This high baseline concentration of anti-HMGCR antibodies is consistent with that in previous studies [19], indicating their role in the pathogenesis of myositis, particularly in the context of immune-mediated muscle damage. After six months of immunosuppressive therapy, combined with bempedoic acid, the average concentration of these antibodies decreased significantly to 220.89 ± 113.37 CU/L, indicating a reduction of over 40%. This decrease was statistically significant (*p* = 0.027), suggesting that the combined therapeutic regimen may have helped mitigate the autoimmune response associated with the disease.

In parallel, CK levels, which are often elevated in myositis due to muscle damage, were assessed as another key marker of disease activity. At the time of myositis onset, the mean CK value was 1278.9 ± 769.39 IU/L, reflecting substantial muscle injury. Following six months of treatment with immunosuppressive agents and bempedoic acid, CK levels decreased significantly to 315.1 ± 157.72 IU/L, indicating a dramatic reduction of over 75% (*p* = 0.001).

Similarly, the average aldolase level at myositis onset was 11.63 ± 2.18 U/L, which is markedly higher than the normal reference range. However, after six months of treatment, the mean aldolase level decreased to 6.61 ± 1.22 U/L (*p* = 0.0001).

Additionally, LDL-C levels were monitored to evaluate the impact of bempedoic acid, which acts primarily to lower LDL-C levels. After six months of treatment, the average LDL-C concentration was found to be 96.1 ± 8.16 mg/dL, which is within the target range for cardiovascular risk management. Although the result was not statistically significant, it underscores the dual benefit of bempedoic acid, not only as a lipid-lowering agent but also as part of a broader therapeutic strategy that addresses both the autoimmune and metabolic aspects of the disease.

To investigate the underlying autoimmune component of the disease further, a comprehensive panel of autoimmune serologic tests was conducted. These tests included markers such as ANAs, anti-Jo-1, U1-snRNP, Sm, anti-Mi2, anti-TIF1γ, anti-MDA5, anti-NXP2, anti-SAE1, anti-Ku, anti-PM-Scl, anti-PL7, anti-PL12, anti-EJ, anti-OJ, and anti-Ro52, all of which were found to be negative in the patient cohort. The absence of these autoantibodies further supports the diagnosis of a distinct form of myositis and suggests that the therapeutic response may not be influenced by the presence of these particular autoantibodies, which are commonly associated with other forms of autoimmune muscle diseases.

## 4. Discussion

According to the European Neuromuscular Center (ENMC) diagnostic criteria, a combination of serum autoantibody positivity, proximal muscle weakness, and elevated serum CK levels is sufficient to establish a diagnosis of anti-HMGCR and anti-SRP myopathies, without the necessity of histopathological confirmation [4]. However, in recent years, the European League Against Rheumatism (EULAR) and the American College of Rheumatology (ACR) have developed myositis classification criteria for IIM that have been widely adopted [20]. These criteria allow for accurate classification by integrating clinical, serological, and pathological features. While primarily designed for research classification, they have also been applied clinically, with several studies demonstrating high sensitivity and specificity for diagnosing IIM [21,22,23,24].

In addition, hyperlipidemia represents a major public health challenge. Approximately 30% of adults in the United States have elevated LDL-C, which doubles their risk of developing cardiovascular disease [25]. HMGCR inhibitors, commonly known as statins, effectively reduce LDL-C levels and have been shown to decrease the incidence of cardiac events by 20 to 44% in both primary and secondary prevention settings [26,27]. The 2013 American College of Cardiology and American Heart Association (ACC/AHA) cholesterol treatment guidelines expanded the number of adults eligible for statin therapy in the U.S. from 43.2 million (37.5%) to 56.0 million (48.6%) [28]. According to the Centers for Disease Control and Prevention (CDC), 26% of adults in the U.S. over 40 years and 48% of those over 75 years report using cholesterol-lowering medications, with statins accounting for 93% of these prescriptions [26].

All of these data highlight the expanding role of statins in clinical practice, given their established efficacy and generally favorable safety profile.

The incidence of statin-induced myopathies reported in randomized controlled trials ranges from 1.5% to 5.0% [29]. Specifically, autoimmune myopathies, including IMNM, are relatively rare, with a prevalence of approximately 9–14 cases per 100,000 adults; among these, only about 6% are positive for anti-HMGCR antibodies [30,31]. Notably, over 90% of cases of anti-HMGCR-associated IMNM occur in adults receiving statin therapy [31].

Research on statin pharmacokinetics suggests that lipophilic statins—such as atorvastatin, fluvastatin, lovastatin, and simvastatin—may carry a higher risk of myotoxicity compared to that for hydrophilic statins like pravastatin and rosuvastatin. This increased risk is thought to arise because lipophilic statins can more readily penetrate skeletal muscle cell membranes due to their fat-soluble nature, thereby increasing the likelihood of muscle-related adverse effects. In contrast, hydrophilic statins, being more water-soluble, have limited muscle cell penetration [32]. Supporting this, Essers et al. identified a stronger association of IMNM with lipophilic statin use [33]. Additionally, drug interactions involving statins being metabolized by cytochrome P450 3A4 (CYP3A4) enzymes may elevate the risk of adverse effects further [34]. IMNM is dose- and type-dependent, and the risk of disease is lower with fluvastatin, pravastatin, and pitavastatin, as these statins are not metabolized by CYP3 A4, reducing the probability of drug interactions. On the other side, risk is influenced by the patient’s age and gender and is higher for lipophilic statins metabolized by CYP3 A4.

Most existing studies in the literature refer to cases treated with atorvastatin [34,35,36,37,38,39,40,41,42,43], although cases have also been reported using rosuvastatin [39,43,44,45], simvastatin [42,44,46,47,48,49], fluvastatin, and pravastatin [43].

In our pilot study, eight patients took atorvastatin, and two were on simvastatin. Moreover, we observed that the mean duration of statin exposure prior to IMNM diagnosis (monitored through the determination of anti-HMGCR antibodies) was 17.1 ± 5.9 months. However, the onset of IMNM can be variable [3]. Indeed, in the Effect of Statins on Muscle Function and Performance (STOMP) study, the median onset of statin-associated myalgias—with CPK levels less than 10 times the upper limit of the normal values—was 35 ± 31 days after starting statin therapy, compared to 61 ± 33 days in the placebo group [50]. In addition, Cham et al. reported a median onset of 14 weeks from statin initiation to IMNM diagnosis, with 85% of cases having definitive or probable causality linked to statin use [51]. Similarly, a large case–crossover study involving 93,831 patients found that most statin myotoxicity cases occurred within the first 12 weeks of treatment but recommended continued monitoring for up to 26 weeks of exposure [52].

In our case series study, at the onset of myositis, patients exhibited high levels of anti-HMGCR antibodies, with an average concentration of 390.93 ± 275.22 CU/L. The presence of such high antibody titers underscores their role not only as diagnostic biomarkers but also as active mediators of disease. So, in this context, MSAs have become essential tools for diagnosing and characterizing IIM, as well as for predicting the treatment response and potentially informing its prognosis [30].

Anti-HMGCR autoantibodies are primarily associated with IMNM, with a prevalence of 44.9%, while the detection levels in other adult-onset forms are 4.4% [53]. The high specificity of the anti-HMGCR autoantibody test has also been demonstrated in patients with non-autoimmune, statin-induced, self-limiting necrotizing myopathy: in one study, 278 patients tested for anti-HMGCR autoantibodies were reported to be negative [53]. Anti-HMGCR autoantibodies are primarily associated with IMNM, with a prevalence of 44.9% in this group, whereas their detection levels in other forms of adult-onset myopathies are much lower, at approximately 4.4% [54].

Tiniakou et al. followed 104 patients with anti-HMGCR myopathy across different age groups at the Johns Hopkins Myositis Center and found that statin exposure occurred in 40% of patients aged 52 years or younger, compared to 89–97% of those older than 52 years. For context, less than half of the U.S. population over 60 years of age is prescribed statins [55]. Among the younger cohort (mean age: 48 vs. 57.4 years), comprising 15 individuals, patients were more likely to be refractory to immunosuppressive therapy and exhibited greater disease severity. The authors hypothesized that this may be due to greater muscle mass in younger patients, which leads to a higher HMGCR antigen load, thereby amplifying the autoimmune response and prolonging the time to remission [55].

Therefore, although they are a valid indicator of anti-HMGCR myopathy, anti-HMCGCR autoantibody tests should be reserved for patients with a high pretest probability (i.e., symptomatic elderly subjects exposed to statins) to improve their positive predictive value and reduce the likelihood of alternative diagnoses [9].

Moreover, in our pilot study, following six months of immunosuppressive therapy combined with bempedoic acid treatment, a significant reduction in anti-HMGCR antibody levels was observed, with their mean values dropping to 220.89 ± 113.37 CU/L—a decrease exceeding 40%. This statistically significant decline (*p* = 0.027) suggests that the combined therapeutic regimen effectively mitigates the autoimmune response underlying the disease. Importantly, this finding supports the hypothesis that immunosuppressive therapy, when paired with bempedoic acid, may exert effects beyond simply controlling muscle inflammation by potentially modulating the autoimmune drivers of IMNM. In parallel, CK levels demonstrated a marked reduction from 1278.9 ± 769.39 IU/L at disease onset to 315.1 ± 157.72 IU/L after treatment, representing a dramatic decrease of over 75% (*p* = 0.001). This substantial drop indicates a significant reduction in ongoing muscle damage and corroborates the clinical improvement observed in the patients. The combined therapy’s impact on CK levels highlights its potency in controlling the musculoskeletal manifestations of IMNM. Similarly, aldolase levels, another sensitive marker of muscle damage, were significantly elevated at the baseline (11.63 ± 2.18 U/L) and declined to 6.61 ± 1.22 U/L following treatment (*p* = 0.0001). This further strengthens the evidence that this treatment regimen not only alleviates clinical symptoms but also effectively reduces biochemical markers of muscle injury, which is critical for disease monitoring and assessing treatment responses. To explore the broader autoimmune profile of the cohort, a comprehensive serologic panel was conducted, including ANAs and myositis-specific antibodies such as anti-Jo-1, anti-Mi2, anti-TIF1γ, anti-MDA5, and others. The absence of these autoantibodies supports the diagnosis of a distinct myositis subtype driven predominantly by anti-HMGCR antibodies. This seronegative profile for other autoantibodies suggests that the therapeutic effects observed are unlikely to be confounded by overlapping autoimmune conditions, reinforcing the specificity of the treatment response.

It is now well established that the treatment of IMNM involves corticosteroids; steroid-sparing immunosuppressants such as methotrexate, mycophenolate mofetil, and azathioprine; and the use of intravenous immunoglobulins (IVIGs) and rituximab in refractory cases [6]. However, the management of hypercholesterolemia following IMNM remains controversial. Some studies suggest that re-challenging patients with statins—often after a temporary suspension and a change in the type of statin—may be feasible. Yet evidence also indicates that worsening disease can still occur even after switching statins [56]. A recent review highlights the potential role of proprotein convertase subtilisin/kexin type 9 (PCSK9) inhibitors as an effective alternative for managing hypercholesterolemia in patients with statin-associated IMNM [57].

In light of current scientific evidence, this is the first case series reporting the use of bempedoic acid in patients affected by IMNM. In our study, LDL-C levels were monitored to evaluate the metabolic impact of bempedoic acid. After six months, their mean LDL-C levels reached 96.1 ± 8.16 mg/dL, within the recommended target range for cardiovascular risk management. Although this result is not statistically significant, likely due to the small number of patients enrolled, it underscores the dual benefit of bempedoic acid in this patient population—lowering lipids while contributing to the management of IMNM. The ability to safely reduce LDL-C without exacerbating muscle symptoms represents a significant clinical advantage, especially given the cardiovascular risks faced by these patients. The Phase 3, double-blind, placebo-controlled CLEAR (Cholesterol Lowering via Bempedoic Acid, an ACL-Inhibiting Regimen) study randomized 345 patients with hypercholesterolemia and a history of intolerance to at least two statins (including one at the lowest available dose) at a 2:1 ratio to receive 180 mg of bempedoic acid or a placebo once daily for 24 weeks. Notably, 93% of the participants reported a history of statin-associated muscle symptoms. Compared with the placebo, bempedoic acid treatment resulted in significant reductions in LDL cholesterol (−17.9%), total cholesterol (−14.8%), apolipoprotein B (−15.0%), and high-sensitivity C-reactive protein (−24.3%). The most common muscle-related adverse event, myalgia, occurred in 4.7% of the patients treated with bempedoic acid versus 7.2% in the placebo group [58]. Moreover, in a study involving 13,970 patients, nearly half reported a history of IMNM. Among these patients, those with a history of IMNM—regardless of randomization to receive bempedoic acid or a placebo—experienced higher rates of treatment discontinuation and more musculoskeletal symptoms, and a greater proportion attempted to restart statin therapy [59].

These findings suggest that patients with a history of IMNM may possess underlying factors that affect their tolerance to statin therapy and may therefore require more tailored clinical management [59]. Although studies have reported only a small percentage of myalgia cases, to date, no instances of autoimmunity induction or necrotizing myopathy triggered by bempedoic acid have been described.

## 5. Conclusions

Our case series pilot study highlights that bempedoic acid can be a valid alternative to statins in intolerant patients who have developed an autoimmune myopathy with anti-HMGCR antibodies. The cost of the drug compared to that of PCSK-9 inhibitors could certainly be an advantage, especially in patients at risk who require primary prevention of cardiovascular events.

Studying larger cohorts of patients treated with bempedoic acid will permit a deeper understanding of the usefulness of different lipid-lowering treatments in different subgroups of patients, including in relation to the heterogeneity of disease patterns.

## Figures and Tables

**Table 1:** Demographic, clinical, and laboratory characteristics of the patients.

I.P	Sex	Age	Time Exposition for Statin (Months)	Statin	Other Lipid-Lowering Agents	Prednisone Dose mg/Day	Immunosuppressant	Timing of Bempedoic Acid Initiation (Months)	LDL-C mg/dL	C-LDL After Bemp. mg/dL	CK IU/L	CK After Bemp. IU/L	Aldolase U/L	Aldolase After Bemp. U/L	Ab-HMGCR CU/L	Ab-HMGCR After Bemp. CU/L
A.G	M	56	22	ATO		25	MTX	3	86	111	1080	263	11	6.7	44.8	41.6
B.I	F	62	25	ATO		20	MTX	3	116	107	998	245	10.8	6.1	170.5	56.8
B.D	M	46	18	ATO	EZE	50	MYC + IgV 40 g	3	101	98	3250	568	15.8	8.1	857	346
C.F	M	56	13	SIM		25	MTX	4	91	89	1231	246	9.8	6.8	739	312
C.M	F	66	14	ATO		25	MTX	4	82	85	988	312	12.2	7.2	667	224
L.L	F	56	9	ATO		12.5	MTX	3	89	97	687	156	11.4	5.1	172.7	156.7
M.M	M	64	15	ATO		25	MYC	4	95	91	1056	345	9.6	6.8	303.9	211
M.F	F	81	27	SIM	EZE	37.5	MYC + IgV 25 g	4	93	96	1874	617	14.6	8.3	435.2	357.8
P.P	F	75	16	ATO		25	MTX	4	94	89	967	241	12.2	6.7	319	314
V.F	F	61	12	ATO		25	MTX	3	101	98	658	158	8.9	4.3	200.2	189
Average		62.3	17.1			27		3.5	94.8	96.1	1279	315.1	11.63	6.61	390.93	220.89
SD		10.08	5.86			10.12		0.53	9.54	8.16	769	157	2.18	1.22	275.22	113.37

## Data Availability

The patient data is contained in their medical records.
